# Radiation metrics for vascular and interventional radiology procedures in a tertiary care institution: A retrospective cohort study over 5-years

**DOI:** 10.15537/smj.2022.43.9.20220194

**Published:** 2022-09

**Authors:** Mohammad Arabi, Ali S. Rajeh, Nasser Alhendi, Khalid T. Alotaibi, Talal A. Yahyan, Khalid Alyousef, Husam Ardah

**Affiliations:** *From the Department of Medical Imaging, Vascular Interventional Radiology, King Abdulaziz Medical City, National Guard Health Affairs, Riyadh, Kingdom of Saudi Arabia.*

**Keywords:** interventional radiology, radiation protection, radiation dosage, diagnostic reference levels

## Abstract

**Objectives:**

To evaluate the radiation metrics from frequently carried out vascular and interventional radiology (VIR) procedures at a tertiary care institution and compare them to international diagnostic reference levels (DRLs).

**Methods:**

A retrospective study of the radiation metrics of VIR-procedures carried out from January 2015 to December 2019. The collected data included age, gender, height and weight, reference point air kerma (mGy), dose area product (DAP; Gy.cm2), and ﬂuoroscopy time (min.) The body mass index (BMI) and peak skin dose were calculated. The study cohort included 8942 adult patients (54.4% male, 45.6% female) with a mean age of 56.96 years and mean BMI of 26.86.

**Results:**

Transjugular intrahepatic portosystemic shunt (TIPS) creation recorded the highest mean ﬂuoroscopy time of 69.41 min., followed by central venous recanalization 39.67min. TIPS creation had the highest mean DAP (1161.16 Gy.cm2), followed by trans arterial chemoembolisation (TACE) (500.63Gy.cm2). TIPS creation was associated with the highest peak skin dose (2766.81mGy), followed by TACE (1588.29mGy). Compared to other studies, TIPS creation and TACE are associated with significantly higher DAP.

**Conclusion:**

Majority of VIR-procedures demonstrate no significant institutional variations in dosimetry compared to other studies. Using these studied values as reference levels may help identifying procedures that need quality control to minimize unnecessary exposures.

Fluoroscopy is increasingly used to guide minimally invasive diagnostic or therapeutic procedures. This exposure to ionizing radiation has inherent potential deterministic or stochastic effects on patients and operators.^1^ Dose optimization can be met effectively by the concept of diagnostic reference levels (DRLs), which was introduced by the International Commission for Radiation Protection (ICRP) as practice guidelines and advised to identify these values in each country or region.2 It has been mandatory since 1997 in the European Union, in addition to the new European directive for 2013/59/Euratom.^3^ Diagnostic reference level is defined as radiation dose levels for typical X-ray examinations for standard-sized patients using standard equipment. National DRLs are set at 75^th^ percentile of the median values of an appropriate quantity in a representative sample of healthcare facilities. Exceeding the DRL for a certain procedure should trigger an investigation of the equipment, procedure protocol, or the operator’s technique. Applying DRLs to interventional procedures is challenging due to multiple factors, including the patient’s size, the complexity of the procedure, and the operator’s experience. Active dosimetry is not only an audit tool, but also optimizes occupational protection for each procedure. The ICRP recommends gathering regional or national dosimetric data for every case of a procedure from a large number of facilities, or alternatively, use a local data set of the dosimetric data of every case of the same procedure carried out at the local facility.^2,4^ Apart from interventional cardiology, there is limited DRLs data for body and neuro interventional procedures, and a paucity of DRLs for pediatric interventional procedures.^4,5^ Recently, the Saudi Food and Drug Authority and Saudi Health Council issued national DRLs for a computed tomography (CT), based on dosimetric data sets from 10 different hospitals in Saudi Arabia. More recently, a local study reported that the radiation dose levels for uterine artery embolization procedures and compared it to published data.^6^

The aim of this study is to participate in establishing the national DRL, reporting the dosimetric data of the most frequently carried out procedures in a tertiary care center and compare it to the reported international data from current literature, the radiation doses in interventional radiology procedure (RAD-IR) study, a radiation doses measurements study carried out for DRLs in 2003, and a recently published study with similar procedure categories to our sample.^7,8^

## Methods

This retrospective review included adult vascular and interventional radiology (VIR) procedures carried out over a period of 5 years from January 2015 to December 2019 at the Department of Medical Imaging, King Abdulaziz Medical City, National Guard Health Affairs, Riyadh, Kingdom of Saudi Arabia. The radiation doses were captured using the dose management software (DoseWatch; GE Healthcare System, Buc, France) that is integrated in the Radiology Information System. The anthropometric data included the height, weight, and body mass index (BMI) in addition to the patients’ age (>14 years) and gender. The dosimetric data included the reference point air kerma (mGy) (known as reference dose, cumulative dose, or cumulative air kerma), dose area product (DAP) (Gy.cm^2^) (known as Kerma area product [KAP]), and ﬂuoroscopy time (minute). The peak skin dose (PSD) was calculated using the reference point air kerma and the following formula: PSD (mGy)=206+0.513*Ka,r [mGy]).^9^

**Figure 1:**
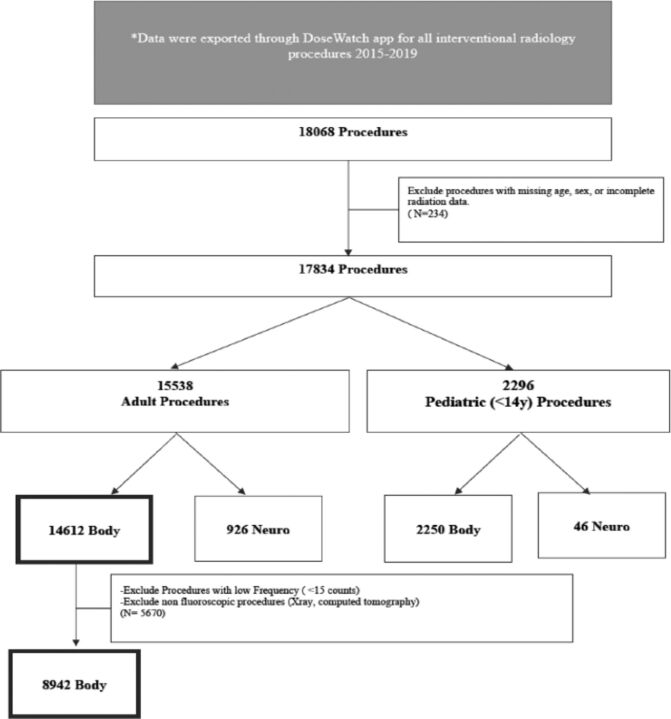
- Flow Chart of the study procedures’ selection.

During the study period, 18068 fluoroscopy-guided interventions were carried out by several consultant interventional radiologists or supervised residents/fellows in training. This analysis did not include neurointerventional (n=972) and pediatric (n=2250) procedures, which will be evaluated separately. Duplicate procedures accessions, procedures with missing dosimetric or demographic data were excluded. Low count procedures (<15) were also excluded, including procedures such as prostate artery embolization, prophylactic bilateral internal iliac artery occlusion, and pulmonary embolectomy (Figure 1).

This study protocol was approved by the Institutional Review Board at King Abdullah International Medical Research Center. The study was carried out in accordance with the principles of the Helsinki Declaration.

Procedures of a similar nature were grouped in one category. For example, the tunneled catheter placement category included new tunneled hemodialysis catheter insertion, in addition to exchange +/- simple fibrin sheath angioplasty, chest port placement, and chemotherapy tunneled catheter insertion. Axial embolization procedures included embolization in the thorax, abdomen or pelvis, and could vary between simple embolization procedures, such as posttraumatic bleeding to a complex embolization of arteriovenous malformation. Arteriovenous dialysis access interventions vary between simple angioplasty to thrombectomy with balloon angioplasty +/- stent placement. Hepatic mapping may include vessel embolization procedures that were not counted under axial embolization. Post liver transplant vascular interventions included hepatic artery, hepatic or portal veins interventions with thrombolysis, and balloon angioplasty with or without stent placement.

All procedures were carried out on one of 5 angiography machines (AlluraXper, Philips Medical systems, Netherlands). All machines are regularly reviewed by the radiation safety officer, and all staff attend a mandatory annual radiation safety course.

The final analysis included 8942 procedures with 4863 (54.4%) male patients and 4079 (45.6%) female patients, with a mean age of 56.96 years (range: 15-117). The mean height was 1.6 meters (range: 1.2-1.91). The mean weight was 68.9 kg (range: 16-214).

The analysis included 22 different VIR-procedures. Peripherally inserted central catheter placement was the most frequently carried out procedure (n=2712, 30.3%), followed by tunneled catheter placement (n=2151, 24.1%), gastrostomy (n=1000, 11.2%), and arteriovenous dialysis access interventions (n=455, 5.1%).

### Statistical analysis.

The categorical data are presented as percentage and frequency, and the continuous variables as mean, median, standard deviation, 25^th^ and 75^th^ percentile, and range. Two independent sample t-test was used to compare the mean values from the current study with the RAD-IR study and Contemporary Interventional Radiology Dosimetry (CIRD) study.^7,8^ Statistical significance was defined as a *p*-value of <0.05. The statistical analysis was carried out on Statistical Analysis System, version 9.4 (Cary, NC, USA) and StatPlus:mac - statistical analysis program for Mac OS. Version v5 (AnalystSoft Inc., WALNUT, CA).

## Results

Transjugular intrahepatic portosystemic shunt (TIPS)-creation recorded the highest mean ﬂuoroscopy time of 69.41±37.82 minutes, followed by central venous recanalization (39.67±40.83 minutes), and uterine fibroid embolization (32.27±15.06 minutes; Table 1). The TIPS-creation had the highest mean DAP (1161.16±840.93 Gy.cm^2^), followed by the trans arterial chemo embolization (500.63±346.02 Gy.cm^2^), and post liver transplant vascular interventions (479.42±394.10 Gy.cm^2^; Table 2). Similarly, the TIPS-creation had the highest mean reference point air kerma of 4.99±3.95 Gy, followed by trans arterial chemo embolization of 2.69±1.81 Gy, and post liver transplant vascular interventions of 2.48±2.35 Gy (Table 3). The TIPS-creation was associated with the highest PSD (2766.81±2024.54 mGy), followed by the trans arterial chemo embolization (1588.29±928.98 mGy), and post liver transplant vascular interventions (1480.66±1204.36 mGy; Table 4).

**Table 1:** - Fluoroscopy time for the analyzed vascualr and interventional procedures.

Study description	Fluoroscopy time (minutes)							
	n	Mean±SD	Median	25th percentile	75th percentile	Range	Minimum	Maximum
Arteriovenous dialysis interventions	455	12.73±15.48	7.55	3.58	16.98	134.63	0.05	134.68
Axial embolization	259	24.15±19.32	19.52	10.60	30.27	121.30	0.27	121.57
Central veins recanalization	46	39.67±40.83	18.06	8.73	60.15	161.43	1.25	162.68
Gastrostomy new insertion	1000	2.22±2.39	1.63	0.87	2.74	29.58	0.00	29.58
Hepatic artery mapping	148	22.76±16.20	18.88	11.25	29.65	80.33	0.03	80.37
IVC filter insertion	244	2.67±4.25	1.58	1.15	2.48	40.73	0.00	40.73
IVC filter removal	116	12.29±22.77	5.11	3.41	9.58	138.20	0.12	138.32
LE angiography/angioplasty	279	22.62±20.46	16.67	7.42	30.40	122.60	0.48	123.08
Lumbar puncture	264	0.44±0.81	0.22	0.10	0.37	6.07	0.00	6.07
Nephrostomy	276	5.09±5.44	3.51	2.08	6.31	44.37	0.08	44.45
Peritoneal dialysis catheter	69	5.56±5.06	4.02	2.22	7.78	29.75	0.03	29.78
PICC line insertion	2712	1.08±2.55	0.45	0.25	0.90	60.40	0.00	60.40
Post liver transplant interventions	25	19.79±14.44	18.87	9.25	27.78	47.13	1.60	48.73
PTC and biliary drainage	335	15.06±15.92	9.57	4.85	20.88	133.05	0.02	133.07
TACE	251	30.29±17.53	25.48	17.73	40.75	123.27	2.38	125.65
TARE	113	11.59±9.95	8.42	4.72	16.27	47.50	1.25	48.75
TIPSS-creation	49	69.41±37.82	62.42	41.25	83.38	182.62	14.00	152.00
TIPSS-revision	55	24.03±32.33	13.93	8.08	31.90	222.10	3.05	225.15
Tunneled catheter placement	2151	1.81±4.58	0.78	0.43	1.65	103.43	0.00	103.43
Uterine fibroid embolization	21	32.27±15.06	29.52	23.68	38.33	70.02	10.33	80.35
Varicocele embolization	19	27.89±13.17	23.40	17.62	36.30	50.60	8.23	58.83
Vertebroplasty	55	7.96±7.70	5.22	2.78	11.42	35.55	0.17	35.72

IVC: inferior vena cava, LE: extremity, PICC: peripherally inserted central catheter, PTC: percutaneous transhepatic cholangiography, TACE: trans arterial chemoembolisation, TARE: Trans-arterial radio-embolization, TIPSS: transjugular intrahepatic portosystemic shunt, n: number, SD: standard deviation

**Table 2:** - Dose area product for the analyzed vascualr and interventional procedures.

Study description	DAP (Gy.cm^2^)							
	n	Mean±SD	Median	25th percentile	75th percentile	Range	Minimum	Maximum
Arteriovenous dialysis access	455	14.29±27.41	6.95	3.25	14.16	355.72	0.19	355.91
Axial embolization	259	324.65±348.60	183.48	77.36	459.17	1814.73	1.45	1816.18
Central veins recanalization	46	115.83±163.10	43.57	20.83	139.08	897.87	9.08	906.96
Gastrostomy new insertion	1000	4.64±7.85	2.48	1.13	5.04	131.55	0.03	131.58
Hepatic artery mapping	148	442.42±390.86	322.25	174.45	567.85	2416.84	0.02	2416.85
IVC filter insertion	244	23.05±39.09	12.29	7.61	23.85	358.36	0.29	358.65
IVC Filter removal	116	38.67±58.92	13.62	8.29	35.22	323.60	3.17	326.77
LE angiography/angioplasty	279	38.29±70.88	15.02	8.83	36.31	781.96	0.80	782.76
Lumbar puncture	264	3.21±10.87	0.49	0.13	1.78	107.65	0.02	79.01
Nephrostomy	276	20.59±35.02	9.73	4.67	21.66	372.73	0.06	372.79
Peritoneal dialysis catheter	69	40.49±49.94	21.83	11.03	54.80	244.63	0.23	244.86
PICC insertion	2712	2.15±10.64	0.79	0.40	1.63	339.36	0.01	339.37
Post liver transplant interventions	25	479.42±394.10	363.71	186.76	650.21	1354.24	88.94	1443.18
PTC & biliary drainage	335	69.48±86.85	38.72	16.81	79.33	542.53	0.01	542.54
TACE	251	500.63±346.02	415.73	235.96	675.51	1831.69	28.18	1859.88
TARE	113	194.17±213.48	107.56	67.82	255.21	1431.99	12.21	1444.19
TIPSS-creation	49	1161.16±840.93	1110.04	473.29	1463.84	3568.33	85.61	3653.94
TIPSS-revision	55	313.49±519.54	132.41	57.10	313.22	3160.12	13.47	3173.59
Tunneled catheter placement	2151	3.47±14.97	1.27	0.61	2.70	476.26	0.02	476.27
Uterine fibroid embolization	21	247.42±351.55	156.14	78.99	259.32	1655.47	37.17	1692.64
Varicocele embolization	19	90.06±76.83	66.22	31.13	124.88	277.09	15.52	292.61
Vertebroplasty	55	76.02±99.94	50.19	14.45	92.26	520.23	1.22	521.45

IVC: inferior vena cava, LE: extremity, PICC: peripherally inserted central catheter, PTC: percutaneous transhepatic cholangiography, TACE: trans arterial chemoembolisation, TARE: Trans-arterial radio-embolization, TIPSS: transjugular intrahepatic portosystemic shunt, N: number, SD: standard deviation

**Table 3:** - Reference point entrance dose for the analyzed vascualr and interventional procedures.

Study description	Reference point entrance dose (air kerma) Gy							
	n	Mean±SD	Median	25th percentile	75th percentile	Range	Minimum	Maximum
Arteriovenous dialysis access	455	0.08±0.25	0.03	0.01	0.07	3.51	0	3.5
Axial embolization	259	1.58±1.75	0.94	0.38	2.13	9.4	0.01	9.4
Central veins recanalization	46	0.75±0.98	0.26	0.13	0.95	3.72	0.03	3.8
Gastrostomy new insertion	1000	0.02±0.03	0.01	0.01	0.02	0.57	0	0.6
Hepatic artery mapping	148	1.87±1.83	1.43	0.65	2.42	14.57	0	15
IVC filter insertion	244	0.08±0.21	0.04	0.03	0.08	2.36	0	2.4
IVC filter removal	116	0.24±0.44	0.08	0.04	0.18	2.31	0.01	2.3
LE angiography/angioplasty	279	0.19±0.36	0.08	0.04	0.19	3.93	0	3.9
Lumbar puncture	264	0.03±0.08	0.01	0	0.02	0.86	0	0.9
Nephrostomy	276	0.12±0.2	0.06	0.03	0.14	1.84	0	1.8
Peritoneal dialysis catheter	69	0.18±0.21	0.1	0.04	0.25	1.09	0	1.1
PICC insertion	2712	0.01±0.06	0	0	0.01	2.36	0	2.4
Post liver transplant interventions	25	2.48±2.35	1.44	0.9	3.47	9.71	0.31	10
PTC & biliary drainage	335	0.45±0.63	0.2	0.08	0.5	4.65	0	4.7
TACE	251	2.69±1.81	2.14	1.37	3.51	8.12	0.17	8.3
TARE	113	0.79±0.84	0.48	0.26	0.97	4.48	0.06	4.5
TIPSS-creation	49	4.99±3.95	4.49	2.3	6.66	19.86	0.59	20
TIPSS-revision	55	1.27±2.08	0.51	0.2	1.41	11.15	0.07	11
Tunneled catheter placement	2151	0.02±0.08	0.01	0	0.01	1.96	0	2
Uterine fibroid embolization	21	1.65±1.89	1.18	0.61	1.99	9	0.21	9.2
Varicocele embolization	19	0.48±0.41	0.39	0.14	0.71	1.52	0.07	1.6
Vertebroplasty	55	0.8±0.97	0.45	0.19	0.92	4.35	0	4.4

IVC: inferior vena cava, LE: extremity, PICC: peripherally inserted central catheter, PTC: percutaneous transhepatic cholangiography, TACE: trans arterial chemoembolisation, TARE: Trans-arterial radio-embolization, TIPSS: transjugular intrahepatic portosystemic shunt, N: number, SD: standard deviation

**Table 4:** - Peak skin dose for the analyzed vascualr and interventional procedures.

Study description	Peak skin dose, mGy (PSD=206+0.513*Ka,r (mGy))							
	n	Mean±SD	Median	25th percentile	75th percentile	Range	Minimum	Maximum
Arteriovenous dialysis access	455	249.29±129.87	221.98	213.18	242.99	1801.54	206.57	2008.11
Axial embolization	259	1015.89±898.36	687.26	400.71	1300.22	4821.79	208.81	5030.60
Central veins recanalization	46	588.70±505.09	336.95	272.48	692.83	1910.13	219.41	2129.53
Gastrostomy new insertion	1000	216.88±17.75	212.09	208.94	218.35	290.24	206.03	496.27
Hepatic artery mapping	148	1166.24±936.46	938.66	539.69	1446.30	7476.71	206.06	7682.77
IVC filter insertion	244	249.25±109.31	224.93	218.83	245.95	1208.56	206.65	1415.21
IVC filter removal	116	327.78±224.23	247.98	225.08	300.59	1183.56	212.49	1396.05
LE angiography/angioplasty	279	305.59±184.04	247.38	227.23	304.75	2016.53	207.84	2224.37
Lumbar puncture	264	219.94±43.13	208.74	206.66	216.35	440.65	206.03	646.68
Nephrostomy	276	269.85±103.13	235.07	220.04	277.30	942.53	206.13	1148.67
Peritoneal dialysis catheter	69	296.35±108.57	258.01	228.43	332.00	559.14	206.29	765.42
PICC insertion	2712	210.27±30.19	207.29	206.68	208.77	1209.19	206.02	1415.21
Post liver transplant interventions	25	1480.66±1204.36	942.30	670.16	1984.39	4982.71	364.17	5346.88
PTC and biliary drainage	335	436.53±321.27	310.80	245.20	462.90	2387.15	206.03	2593.18
TACE	251	1588.29±928.98	1302.68	911.02	2005.58	4165.06	291.64	4456.70
TARE	113	609.01±430.09	451.63	338.81	702.35	2296.20	235.01	2531.21
TIPSS-creation	49	2766.81±2024.54	2508.44	1385.41	3620.99	10186.09	509.22	10695.31
TIPSS-revision	55	858.23±1067.33	470.17	310.85	929.29	5720.40	242.75	5963.14
Tunneled catheter placement	2151	214.90±38.73	208.81	207.41	212.45	1004.54	206.03	1210.57
Uterine fibroid embolization	21	1054.61±970.29	809.97	519.93	1225.64	4618.18	314.64	4932.82
Varicocele embolization	19	450.89±208.87	408.27	277.97	569.99	781.70	240.40	1022.10
Vertebroplasty	55	616.82±496.87	434.62	303.34	676.23	2230.49	208.39	2438.88

IVC: inferior vena cava, LE: extremity, PICC: peripherally inserted central catheter, PTC: percutaneous transhepatic cholangiography, TACE: trans arterial chemoembolisation, TARE: Trans-arterial radio-embolization, TIPSS: transjugular intrahepatic portosystemic shunt, N: number, SD: standard deviation

Compared to the CIRD and RAD-IR studies, vascular access procedures had comparable fluoroscopy time, DAP, and reference point air kerma. However, TIPS-creation and trans arterial chemoembolisation (TACE) were associated with significantly higher fluoroscopy time, DAP, and reference point air kerma in our institution. The mean fluoroscopy time for nephrostomy and gastrostomy procedures was significantly shorter in this study compared to the reported values. Dose area product was also significantly lower for nephrostomy, inferior vena cava filter placement, and gastrostomy procedures. Other differences and variations in radiation exposure parameters are illustrated in Table 5.

**Table 5:** - Comparison of the radiation doses between the current study and the CIRD and RAD-IR studies.

Procedure	Study	n	Time		DAP		Reference point air kerma	
			Mean±SD	*P*-values	Mean±SD	*P*-values	Mean±SD	*P*-values
TIPSS-creation	Current study	49	69.41±37.82		1161.20±840.90		4.99±3.95	
	CIRD	120	49.10±16.00	0.0006	429.20±244.80	<0.0001	2.00±1.42	<0.0001
	RAD-IR	135	38.70±27.27	<0.0001	335.40±264.60	<0.0001	2.04±1.65	<0.0001
TACE	Current study	251	30.29±17.53		500.60±346.00		2.69±1.81	
	CIRD	395	18.80±12.50	<0.0001	354.60±78.60	<0.0001	1.75±0.44	<0.0001
	RAD-IR	126	16.80±12.03	<0.0001	282.30±171.40	<0.0001	1.41±1.09	<0.0001
PTC/biliary drainage	Current study	335	15.06±15.92		69.48±86.85		0.45±0.63	
	CIRD	101	11.50±7.10	0.0016	17.30±13.20	<0.0001	0.18±0.07	<0.0001
	RAD-IR	123	23.60±20.94	<0.0001	70.64±68.86	0.8818	0.91±1.00	<0.0001
Nephrostomy	Current study	276	5.09±5.44		20.59±35.02		0.12±0.20	
	CIRD	778	10.40±18.10	<0.0001	15.20±13.15	<0.0001	0.13±1.41	<0.0001
	RAD-IR	143	13.68±12.04	<0.0001	34.32±51.60	0.0047	0.42±0.71	<0.0001
IVC filter placement	Current study	244	2.67±4.25		23.05±39.09		0.08±0.21	
	CIRD	278	3.80±17.80	0.306	36.80±29.30	<0.0001	0.14±1.65	0.5781
	RAD-IR	279	2.80±2.56	0.6779	44.51±31.62	<0.0001	0.17±0.13	<0.0001
PICC	Current study	2712	1.08±2.55		2.15±10.64		0.01±0.06	
	CIRD	755	2.00±26.40	0.3374	1.80±3.10	0.1377	0.01±0.19	0.9640
Tunneled catheters	Current study	2151	0.02±0.08		3.47±14.97		0.02±0.08	
	CIRD	304	0.02±0.96	0.9616	4.20±3.40	0.0543	0.02±0.96	0.9616
Gastrostomy	Current study	1000	2.28±2.39		4.64±7.85		0.02±0.03	
	CIRD	1006	7.00±17.50	<0.0001	6.60±27.60	0.0308	0.03±0.37	0.2716
Embolization	Current study	259	24.15±19.32		324.65±348.60		1.57±1.75	
	CIRD	188	25.30±11.40	0.4327	298.50±29.10	<0.0001	1.62±0.38	<0.0001

DAP: dose area product, IVC: inferior vena cava, PICC: peripherally inserted central catheter, PTC: percutaneous transhepatic cholangiography, TACE: trans arterial chemoembolisation, TIPSS: transjugular intrahepatic portosystemic shunt, CIRD: Contemporary Interventional Radiology Dosimetry, RAD-IR: radiation doses in interventional radiology procedure N: number, SD: standard deviation

There was very strong positive correlation between reference point air kerma and DAP (R=0.93; *p*<0.0001). There was moderate positive correlation between fluoroscopy time and DAP (R=0.60; *p*<0.0001), reference point air kerma (R=0.66; *p*<0.0001), and PSD (R=0.60; *p*<0.0001). There was a very weak positive correlation between BMI with DAP (R=0.08; *p*<0.0001), reference point air kerma (R=0.08; *p*<0.0001), and fluoroscopy time (R=0.04; *p*<0.0001). Noteworthy, the mean BMI for TIPS was 28.1 kg/m^2^, which correlated very weakly with the recorded doses (PSD of 0.09 and DAP of 0.12). Similarly, the TACE patients had a mean BMI of 28.9 kg/m^2^ that correlated weakly (PSD of 0.29 and DAP of 0.34; <0.2: very weak correlation; 0.21-0.4: weak correlation; 0.41-0.7: moderate correlation; 0.71-0.9: strong correlation; and >0.9: very strong correlation).

## Discussion

This study established dosimetric data for the most frequently carried out VIR-procedures in a tertiary care center in Saudi Arabia. Society of Interventional Radiology (SIR) and ICRP recommend the implementation of DRLs to optimize dose monitoring and tracking in patients undergoing VIR-procedures.^3,9^ There is a substantial variation in the dosimetric data related to differences in the operating equipment, operator experience, patient’s habitus, clinical condition, and the complexity of interventions. This is reflected in literature demonstrating a spectrum of radiation exposure for similar interventions.^7,8,10-17^ The complexity and wide variability of radiation exposure in VIR necessitates a review of a large number of procedures to establish institutional and national DRLs that can be used to monitor and optimize the quality of radiation exposure. Miller et al^14^ proposed radiation levels for a reference dose, kerma-air product and fluoroscopy times for several interventional procedures based on the RAD-IR study and a comparison with published European dose data. Ruiz-Cruces et al^10^ and Heilmaier et al^16^ proposed categorizing VIR-procedures in 3 different levels of complexity with corresponding diagnostic reference levels. However, there persists a significant difference in the recommended thresholds for the same complexity level. For example, the reference level of KAP for a complex biliary drainage procedure is proposed at 141 Gy.cm2 and 62.2 Gy.cm2.^10,16^ This compares to a mean of 70 Gy.cm^2^ and 75^th^ percentile of 79.3 in our study irrespective of the patients’ habitus and procedure complexity in our dataset. Transjugular intrahepatic portosystemic shunt and TACE in the current study were associated with significantly higher mean reference levels compared to the CIRD and RAD-IR studies.^7,8,13^ This may be explained by the low volume and higher complexity of TIPS procedures in our training institution. The TACE procedures were carried out as super selective as possible, and frequently mandated a cone-beam CT for lesion localization, which could have contributed to the additional exposure. The large standard deviation for DRLs of TIPS and TACE indicates the significant variability in complexity and required radiation exposure.

Comparing our data with the local study for uterine artery embolization (UAE), the median DAP value was higher at 347 Gy.cm2 for the UAE procedure, compared to our median DAP at 156.14 Gy.cm^2^.^6^

The SIR guidelines suggested DRL thresholds for intra procedural notifications and set the following thresholds to prompt patient follow-up after VIR-procedures: a PSD of 3000 mGy, reference point air kerma of 5000 mGy, KAP of 500 Gy.cm^2^, or a fluoroscopy time more than 60 minutes.^9^ Although these values are not restrictive and should not be regarded as endpoints for procedure termination, the currently reported values may be used by peer institutions and authorities with a similar population, and may be incorporated in quality improvement measures and dose management software.

### Study limitations

This study is limited to an adult population, excluding pediatric patients and neuro interventions, unlike the CIRD and RAD-IR studies.^7,8,13^ These will be reported separately given the inherent differences in interventions and patients as well as the operators. Although there was a very weak positive correlation between the BMI and the reported DRLs, the adult population was largely overweight with a mean BMI of 27 kg/m^2^, which requires additional subgroup analysis to determine the correlation between BMI and DRLs for individual procedures. This study also reports the PSD, which is regarded as the main tissue of concern in interventional procedures and can be used as a threshold to trigger patient follow-up.

In conclusion, the majority of these procedures demonstrate no significant institutional variation in dosimetry, when compared to other studies. However, optimization of radiation precautions is paramount to maintain the exposure as low as reasonably achievable, particularly in TIPS-creation and embolization procedures that are associated with the highest radiation exposure.
